# c-Abl kinase-mediated phosphorylation of γ-tubulin promotes γ-tubulin ring complexes assembly and microtubule nucleation

**DOI:** 10.1016/j.jbc.2022.101778

**Published:** 2022-02-26

**Authors:** Guang-Fei Wang, Qincai Dong, Yu Bai, Jing Gu, Qingping Tao, Junjie Yue, Rui Zhou, Xiayang Niu, Lin Zhu, Caiwei Song, Tong Zheng, Di Wang, Yanwen Jin, Hainan Liu, Cheng Cao, Xuan Liu

**Affiliations:** 1 Department of Genetic Engineering, Beijing Institute of Biotechnology, Beijing, China; 2 Institutes of Physical Science and Information Technology, Anhui University, Hefei, China

**Keywords:** centrosome, γ-tubulin ring complex, microtubule nucleation, c-Abl, γ-tubulin, phosphorylation, CDK5RAP2, cyclin-dependent kinase 5 regulatory subunit associated protein 2, GCPs, γ-tubulin complex proteins, MTOC, microtubule organizing center, PCM, pericentriolar material, γTuRC, γ-tubulin ring complex, γTuSC, γ-tubulin small complex

## Abstract

Cytoskeletal microtubules (MTs) are nucleated from γ-tubulin ring complexes (γTuRCs) located at MT organizing centers (MTOCs), such as the centrosome. However, the exact regulatory mechanism of γTuRC assembly is not fully understood. Here, we showed that the nonreceptor tyrosine kinase c-Abl was associated with and phosphorylated γ-tubulin, the essential component of the γTuRC, mainly on the Y443 residue by *in vivo* (immunofluorescence and immunoprecipitation) or *in vitro* (surface plasmon resonance) detection. We further demonstrated that phosphorylation deficiency significantly impaired γTuRC assembly, centrosome construction, and MT nucleation. c-Abl/Arg deletion and γ-tubulin Y443F mutation resulted in an abnormal morphology and compromised spindle function during mitosis, eventually causing uneven chromosome segregation. Our findings reveal that γTuRC assembly and nucleation function are regulated by Abl kinase-mediated γ-tubulin phosphorylation, revealing a fundamental mechanism that contributes to the maintenance of MT function.

Microtubules (MTs) are one of the most important cytoskeleton elements and play crucial roles in cell shape maintenance, organelle movement, and cell division. The minus ends of MTs are nucleated from and anchored to the MT organizing center (MTOC), a specialized structure responsible for MT assembly from the centrosome or Golgi apparatus during interphase and from the spindle during mitosis (1, 2). In mammalian cells, the centrosome is the major organelle that functions as an MTOC and consists of a pair of centrioles with an orthogonal orientation to each other, which are embedded in a tightly clustered electron-dense pericentriolar material (PCM) (1). The PCM is an organized lattice-like structure containing more than 100 different proteins (3), including several scaffold proteins, such as pericentrin (PCNT), CDK5RAP2, Cep152, Cep192, and CPAP, effector proteins, such as γ-tubulin, and other kinases or phosphatases (4). Scaffold proteins are localized at the core matrix of the PCM, while effector proteins serve as sites of centrosomal MT nucleation (4).

The γ-tubulin ring complex (γTuRC), which functions as an MT nucleator in the PCM, is an ∼2.2 MDa complex composed of γ-tubulin and several γ-tubulin complex proteins (GCPs, GCP2∼6) belonging to a conserved protein family (5). The γ-tubulin small complex (γTuSC) is the elemental unit of the γTuRC, consisting of two molecules of γ-tubulin and one molecule each of GCP2 and GCP3 (6). The γTuRC is composed of two highly asymmetric but similarly sized halves: a core of four γTuSCs and an arrangement of different γTuSC-like subunits (GCP4/5 and GCP4/6) capped by a terminal γTuSC that together form a large and diverse binding surface for regulatory factors (7, 8). MT nucleation at MTOCs requires a regulatory mechanism to recruit γTuRCs. The regulators might be PCM components, such as PCNT, CDK5RAP2, and NEDD1, which always function as γTuRC-associated proteins (9). Beyond initiating MT assembly in the PCM, γTuRCs also play essential roles in the nucleation of MT triplets derived from the interior of the centriole and in supporting the structure of the centriole (10, 11, 12).

Abl nonreceptor tyrosine kinase plays important roles in cell proliferation, differentiation, adhesion, migration, and morphogenesis (13). The N-terminus of c-Abl contains Src homology 3 (SH3), SH2, and tyrosine kinase domains (14, 15), while the C-terminus contains F-actin-binding domains and nuclear localization signals (NLSs) (16, 17), allowing it to shuttle between the nucleus and cytoplasm. Abl-related gene (Arg), the other member of the Abl kinase family, shares a redundant function with c-Abl. Mice with *abl1* gene deletion displayed increased perinatal mortality, a runted phenotype, and abnormal thymus, spleen, head, and eye development (18, 19). Moreover, embryos deficient in both *abl1* and *abl2* showed defects in neurulation and died before 11 days post coitum (20). Both c-Abl and Arg bind microfilaments using their F-actin binding domains, which regulate cell morphology and cytoskeletal dynamics (16, 21). Moreover, Arg can directly bind growing MTs through its MT-binding domain to promote MT polymerization and stability, as well as the interaction between MTs and F-actin (22, 23). c-Abl lacks an MT-binding domain, but it can regulate MT stability by phosphorylating MT regulatory proteins, such as CLASP, an MT plus-end tracking protein (24). Although these data reveal some mechanisms in MT dynamic regulation by Abl family kinases, how MT nucleation at MTOC is regulated by the kinases is not fully understood.

In this study, we showed that c-Abl associated with and phosphorylated γ-tubulin on tyrosine 443 (Y443). Through this phosphorylation, c-Abl regulated the nucleation of centrosomal and Golgi apparatus-derived MTs. Phosphorylation defects of this site on γ-tubulin by c-Abl impaired the assembly of both MTs and PCM in interphase and changed the spindle structure in mitosis by impairing γTuRC assembly.

## Results

### c-Abl associates with γ-tubulin

**Figure 1:**
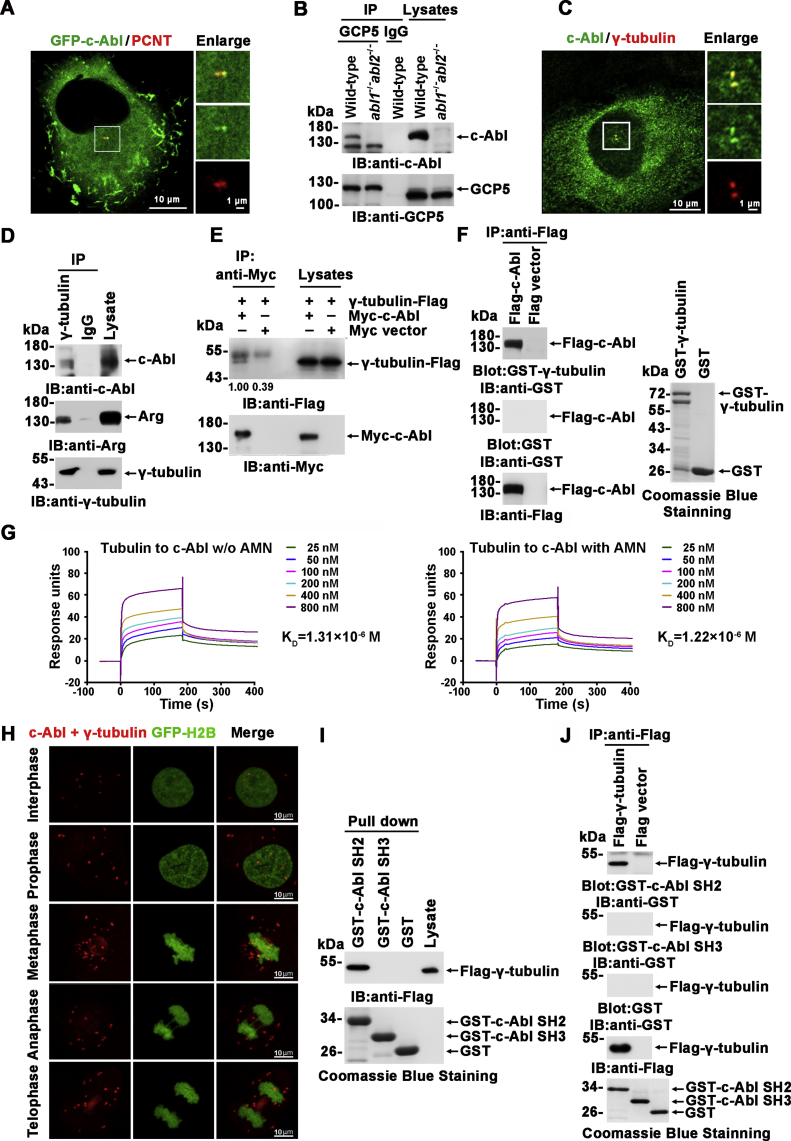
**c-Abl binds γ-tubulin.***A*, immunofluorescence microscopy of anti-PCNT (*red*)-stained MCF-7 cells expressing GFP-c-Abl. The centrosomes are shown in the insets and are enlarged at right. *B*, Anti-GCP5 and IgG immunoprecipitates prepared from wild-type or *abl1*^*−/−*^*abl2*^*−/−*^ U2OS cells were subjected to SDS-PAGE and immunoblotting using the indicated antibodies. *C*, U2OS cells infected with Myc-c-Abl-expressing adenovirus were stained with anti-c-Abl (*green*) and anti-γ-tubulin (*red*). The centrosomes are shown in the insets and are enlarged at right. *D*, Anti-γ-tubulin and IgG immunoprecipitates prepared from MCF-7 cells were subjected to SDS-PAGE and immunoblotting using the indicated antibodies. *E*, lysates of HEK293T cells cotransfected with the indicated plasmids were immunoprecipitated with anti-Myc and analyzed by immunoblotting. *F*. Anti-Flag immunoprecipitates prepared from HEK293T cells transfected with Flag-c-Abl were resolved by SDS-PAGE, transferred onto PVDF membranes, and blotted with GST-γ-tubulin or GST as a control (*left panel*). GST-γ-tubulin and GST were assessed by Coomassie blue staining (*right panel*). *G*, direct interaction between c-Abl and γ-tubulin was assessed by SPR in the presence or absence of AMN107. *H*, *in situ* PLA with anti-c-Abl and anti-γ-tubulin antibodies in HeLa cells stably expressing GFP-H2B. Duolink signals are visualized in *red*, and nuclei are represented by GFP-H2B. *I*, lysates from HEK293T cells expressing Flag-γ-tubulin and Myc-c-Abl were incubated with soluble GST fusion proteins or GST alone. The immunoprecipitates fractions were analyzed by immunoblotting with anti-Flag antibody. GST-tagged proteins were assessed by Coomassie blue staining. *J*, Anti-Flag immunoprecipitates prepared from HEK293T cells expressing Flag-γ-tubulin were subjected to far-western blotting analysis with GST-c-Abl SH2, GST-c-Abl SH3 or GST as described in (*F*). The IP assays or Pull-down assays were repeated at least twice.

In our previous study, to investigate the mechanism of Abl family kinases in the regulation of the MT cytoskeleton, we observed that GFP-c-Abl colocalized with PCNT in the centrosome in MCF-7 cells (Fig. 1*A*). Next, c-Abl was confirmed to be present in anti-GCP5 (but not in control IgG) immunoprecipitates prepared from U2OS cell lysates (Fig. 1*B*), which suggested that c-Abl might be associated with γTuRC. As a control, no c-Abl band was observed in the immunoprecipitates prepared from U2OS cells in which nonsense mutations were introduced in both *abl1* and *abl2* alleles by CRISPR/Cas9 nickase-mediated mutagenesis (Fig. S1, *A* and *B*). Further, c-Abl was observed to colocalize with γ-tubulin, the most important protein for MT nucleation and a component of the γTuRC, in centrosomes (Fig. 1*C*). Moreover, endogenous c-Abl and Arg were demonstrated to coimmunoprecipitate with γ-tubulin (Fig. 1*D*), and overexpressed Myc-c-Abl was observed to interact with γ-tubulin-Flag (Fig. 1*E*). In addition, Flag-c-Abl expressed in HEK293T cells was immunoprecipitated with an anti-Flag antibody, fractioned by SDS-PAGE, and transferred to PVDF membranes. Blotting with GST-γ-tubulin or GST alone demonstrated that Flag-c-Abl directly interacted with γ-tubulin but not GST (Fig. 1*F*). The interaction was further demonstrated by surface plasmon resonance (SPR) analysis, and γ-tubulin was found to bind similarly with c-Abl in the presence (K_D_ 1.22 × 10^−6^ M) or absence (K_D_ 1.31 × 10^−6^ M) of c-Abl inhibitor AMN107, in a buffer without ATP (Fig. 1*G*). Moreover, an *in situ* PLA showed that endogenous c-Abl was associated with γ-tubulin in the cell during interphase, and the association was potentiated in mitosis. Notably, associations in the spindle and cytokinetic furrow were observed during metaphase and cytokinesis, respectively (Fig. 1*H*).

To further characterize the interaction between c-Abl and γ-tubulin, lysates from cells expressing Flag-γ-tubulin and Myc-c-Abl were incubated with GST-c-Abl SH2-, GST-c-Abl SH3-, or GST-conjugated agarose beads. Analysis of the absorbates with anti-Flag showed that Flag-γ-tubulin interacted with c-Abl SH2 but not SH3 (Fig. 1*I*). Far-western analysis also demonstrated that Flag-γ-tubulin directly interacted with c-Abl SH2 but not SH3 (Fig. 1*J*). Collectively, these findings indicated that c-Abl physically associated with γ-tubulin in the cell.

### c-Abl phosphorylates γ-tubulin

**Figure 2:**
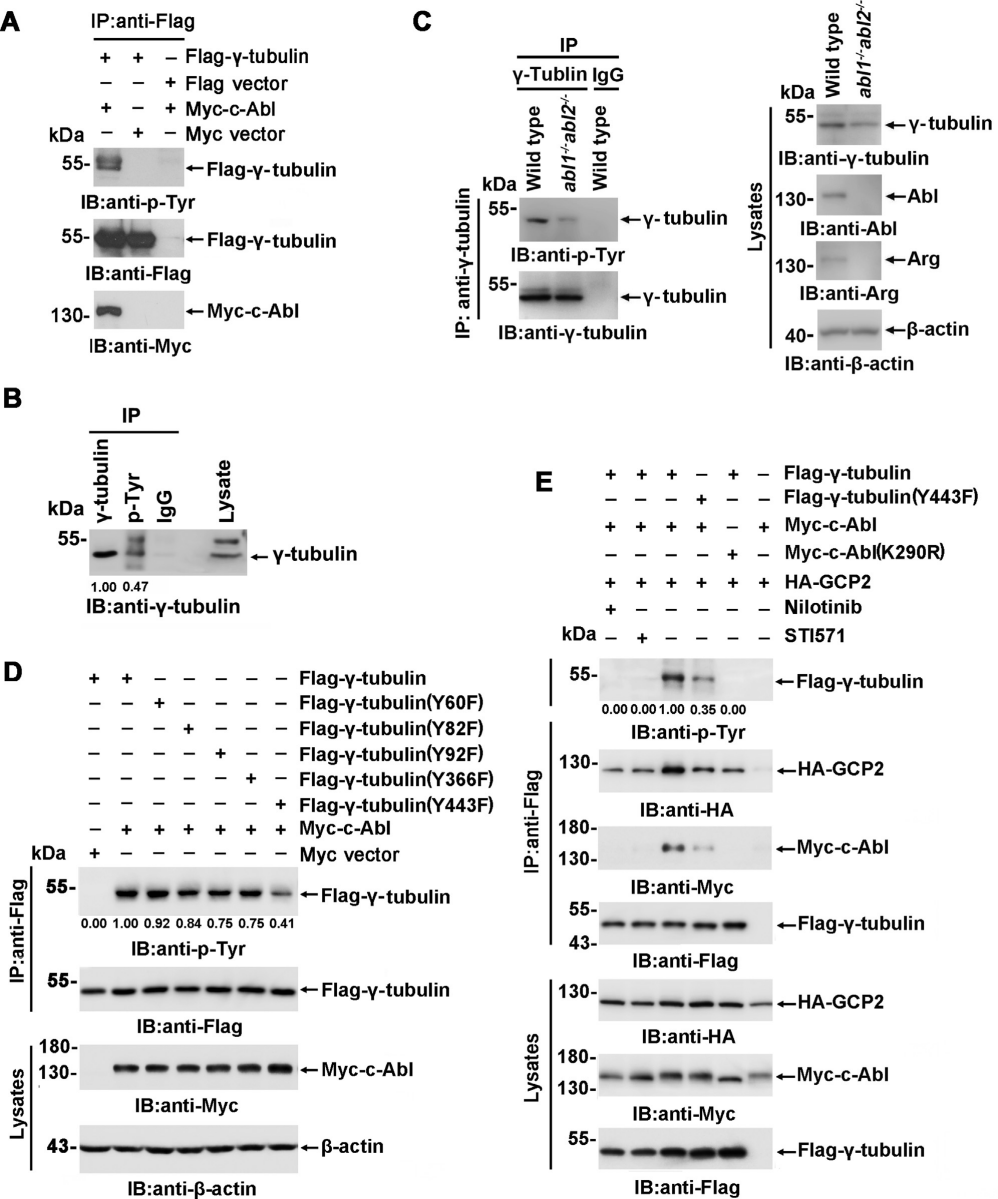
**c-Abl phosphorylates γ-tubulin.***A*, Anti-Flag immunoprecipitates prepared from HEK293T cells transfected with the indicated plasmids were subjected to SDS-PAGE and immunoblotting using the indicated antibodies. *B*, Anti-γ-tubulin, anti-p-Tyr and IgG immunoprecipitates prepared from MCF-7 cells were analyzed with anti-γ-tubulin. The intensities of the bands were analyzed using ImageJ. *C*, Anti-γ-tubulin immunoprecipitates from MCF-7 cells or *abl1/abl2* DKO cell lines were subjected to SDS-PAGE and immunoblotting with the indicated antibodies. *D* and *E*, Anti-Flag immunoprecipitates prepared from HEK293T cells transfected with the indicated plasmids were subjected to SDS-PAGE and immunoblotting using the indicated antibodies. Cells also treated with or without nilotinib (10 μM) or STI571 (10 μM) in *E*. The IP assays or Pull-down assays were repeated at least twice.

The observation that γ-tubulin binds c-Abl SH2 suggested that γ-tubulin was a substrate of c-Abl. To demonstrate the c-Abl-mediated phosphorylation of γ-tubulin, Flag-γ-tubulin was coexpressed with Myc-c-Abl or Myc vector. Analysis of the anti-Flag immunoprecipitates with anti-p-Tyr demonstrated that Flag-γ-tubulin was phosphorylated by c-Abl (Fig. 2*A*). Moreover, MCF-7 cell lysates were immunoprecipitated with anti-γ-tubulin or anti-p-Tyr, and the analysis of the immunoprecipitates with anti-γ-tubulin showed that a substantial proportion of γ-tubulin (∼47%) in the cell was tyrosine phosphorylated (Fig. 2*B*). The phosphorylation level of γ-tubulin was analyzed in *abl1*^*−/−*^*abl2*^*−/−*^ double knockout cells and wild-type cells. The γ-tubulin phosphorylation was significantly diminished in *abl1/abl2*-depleted cells. Next, LC-MS/MS was carried out to reveal the potential phosphorylation sites in γ-tubulin in the presence of Myc-c-Abl, and five potential tyrosine sites were identified (Fig. S2). Then, wild-type Flag-γ-tubulin or Flag-γ-tubulin bearing the Y→F mutations was cotransfected with Myc-c-Abl, and the results indicated that the mutation in Flag-γ-tubulin(Y443F) obviously compromised the phosphorylation of γ-tubulin (Fig. 2*D*). Then, the specificity of c-Abl-mediated phosphorylation of γ-tubulin was further demonstrated by the observation that little if any phosphorylation was mediated by dominant negative c-Abl(K290R) or c-Abl in the presence of the c-Abl inhibitor nilotinib or STI571. Flag-γ-tubulin(Y443F) also showed compromised phosphorylation (Fig. 2*E*). There’s no detectable c-Abl in the anti-Flag immunoprecipitants in the presence of c-Abl inhibitor nilotinib or STI571 or by expressing dominant negative c-Abl (K290R). Collectively, these results demonstrated that c-Abl mediated the phosphorylation of γ-tubulin, primarily on Y443 near its C-terminus, which is important for γTuRC assembly, and c-Abl-mediated γ-tubulin phosphorylation significantly potentiated the c-Abl/γ-tubulin interaction.

### c-Abl promotes γTuRC assembly

**Figure 3:**
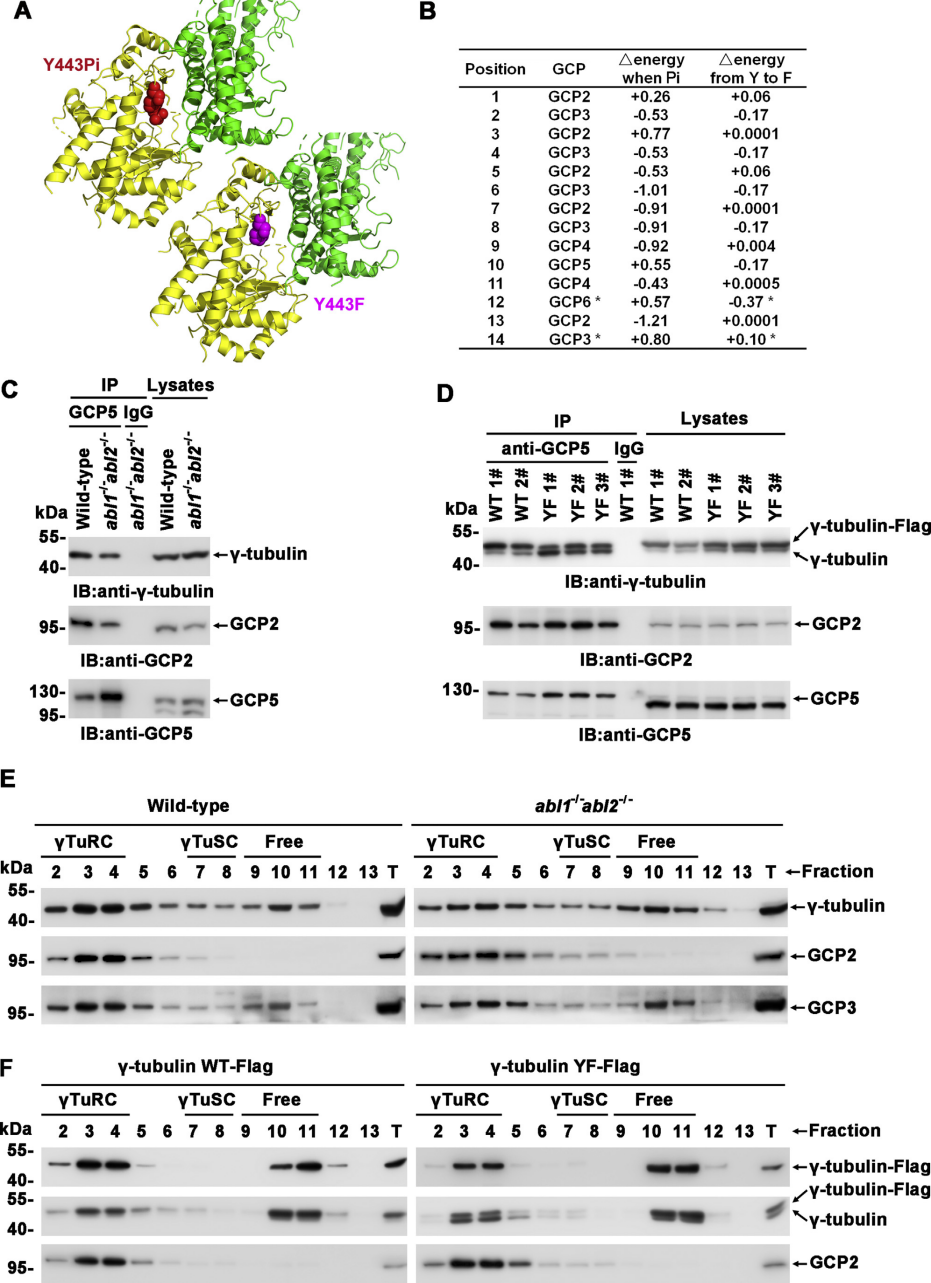
**c-Abl-mediated γ-tubulin phosphorylation potentiates γTuRC assembly.***A* and *B*, interactions between GCP2 and γ-tubulin bearing phosphorylated tyrosine at Y443 or not were calculated by FoldX, and the structural illustrations generated by PyMOL are presented (*A*). γ-tubulin is colored *yellow*, and GCP2 is colored *green*. The phosphorylated Y443 and the Y443F mutant are highlighted in *red* and *magenta*, respectively, and shown as spheres. The changes in binding free energy (kcal/mol) in γ-tubulin-GCP interactions are presented in (*B*), Pi: phosphorylation. *C*, Anti-GCP5 and IgG immunoprecipitates prepared from wild-type or *abl1*^*−/−*^*abl2*^*−/−*^ MCF-7 cells were subjected to SDS-PAGE and immunoblotting. *D*, Anti-GCP5 and IgG immunoprecipitates prepared from U2OS cells stably expressing γ-tubulin-Flag or γ-tubulin(Y443F)-Flag were subjected to SDS-PAGE and immunoblotting using the indicated antibodies. *E* and *F*, lysates from wild-type and *abl1*^*−/−*^*abl2*^*−/−*^ MCF-7 (*E*) or U2OS cells stably expressing γ-tubulin-Flag or γ-tubulin(Y443F)-Flag were fractionated by gel filtration chromatography, and the fractions were immunoblotted with antibodies as noted. T: 5% (v/v) total lysate.

Overall, less GCP2 was present in γ-tubulin immunoprecipitates in the presence of c-Abl/Arg selective inhibitors, after coexpression with dominant negative c-Abl(K290R), or when tyrosine Y443 was mutated to F (Fig. 2*E*), which suggested that c-Abl-mediated phosphorylation of γ-tubulin is involved in γTuRC assembly. The binding energy between γ-tubulin and GCPs was then calculated based on the 3D structure of the γTuRC with FoldX software (8). Phosphorylation at γ-tubulin Y443 generally caused a decrease in the binding energy with the coupled CGPs at most positions on the γTuRC ring (Fig 3, *A* and *B*) and resulted in an increased interaction between γ-tubulin and GCPs (Fig. 3*A*). However, no significant changes in the binding free energies (−0.5 kcal/mol < binding free energy <0.5 kcal/mol) were observed in any γ-tubulin–GCP interactions when Y443 was mutated to F, suggesting that the Y→F mutation itself did not influence the γ-tubulin–GCP interaction (Fig. 3*B*), and it is reasonable to use the Y443F mutant to mimic γ-tubulin bearing nonphosphorylated Y443. In support of the molecular modeling findings, less γ-tubulin was present in anti-GCP5 immunoprecipitates in c-Abl/Arg-depleted MCF-7 cells than in wild-type cells (Fig. 3*C*). Then, γTuRCs in the lysates of stable cell clones expressing Flag-tagged wild-type γ-tubulin or γ-tubulin(Y443F) at similar levels were immunoprecipitated with anti-GCP5, and significantly more wild-type γ-tubulin-Flag was observed to be incorporated into γTuRCs than γ-tubulin(Y443F), as shown by the ratio of ectopically expressed γ-tubulin-Flag and endogenous γ-tubulin (Figs. 3*D* and S3*A*). Moreover, γTuRCs, γTuSCs, or free subunits were fractioned by Superose-6 gel filtration chromatography, and less γTuRC was observed in c-Abl/Arg-depleted cells (Figs. S3*B*, 3*E* and S3*D*), in cells treated with nilotinib (Fig. S3, *C* and *E*), and in cells stably expressing γ-tubulin(Y443F) (Figs. 3*F* and S3*F*), as indicated by the levels of γ-tubulin and GCPs and the ratio of γ-tubulin-Flag:endogenous γ-tubulin. All of the above data suggested that Y443 phosphorylation of γ-tubulin mediated by c-Abl/Arg kinases potentiated the formation of the γTuRC.

### c-Abl regulates PCM formation and centrosome maturation

**Figure 4:**
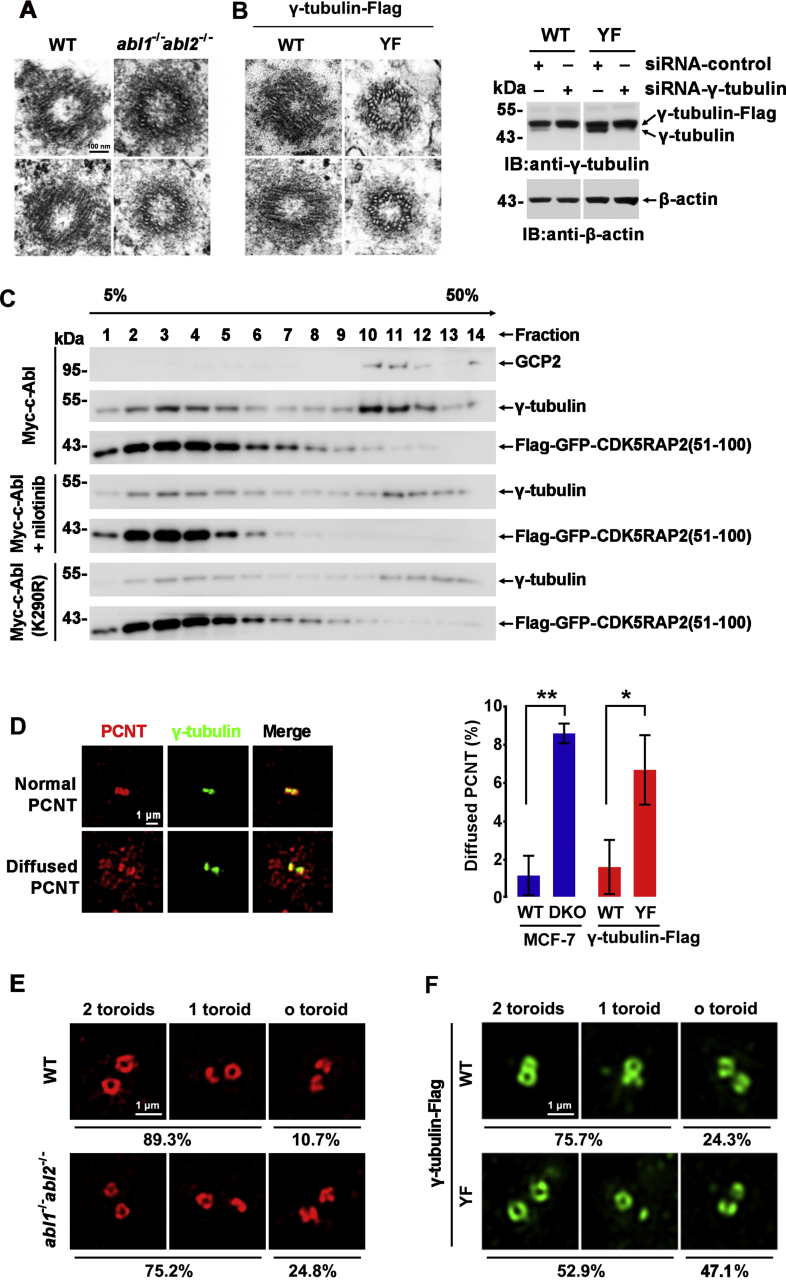
**c-Abl-mediated γ-tubulin phosphorylation promotes centrosome assembly.***A* and *B*, TEM micrographs of centrosomes in wild-type or abl1^−/−^abl2^−/−^ U2OS cells (*A*, cell with visible centrioles: WT-0/5 *versus*. KO-4/5) and U2OS cells stably expressing γ-tubulin-Flag or γ-tubulin(Y443F)-Flag in which endogenous γ-tubulin expression was suppressed by RNAi (*B*, cell with visible centrioles: WT-0/13 *versus*. Y443F mutant-13/15). *C*, cell lysates from HEK293T cells cotransfected with Flag-GFP-CDK5RAP2(51–100) and the indicated plasmids and treated with or without nilotinib (10 μM) were subjected to immunoprecipitation using anti-Flag beads, after which the Flag peptide eluates were fractionated by sucrose gradient centrifugation, and the fractions were immunoblotted with the indicated antibodies. *D*, 3D-SIM micrographs of at least 40 cells stained with anti-PCNT (*red*) and anti-γ-tubulin. Cells with normal or diffused PCNT around the centrosome (*left*) and the percentages (mean±SD of cells with diffused PCNT in three independent experiments, *right*) are shown. *E* and *F*, 3D-SIM micrographs of wild-type (122 cells) and *abl1*^*−/−*^*abl2*^*−/−*^ U2OS cells (117 cells) (*E*) and U2OS cells stably expressing γ-tubulin-Flag (71 cells) or γ-tubulin(Y443F)-Flag (71 cells) (*F*) stained with anti-PCNT. The percentages of the cells bearing 0, 1, or 2 toroidal PCNT are shown at the bottom of each panel.

The PCM of the centrosome provides a kinetically favorable site for cellular MT nucleation. MCF-7 cells deficient in c-Abl/Arg showed a significantly lower PCM density by TEM than wild-type cells (Fig. 4*A*). Stable U2OS clones expressing γ-tubulin(Y443F)-Flag also had decreased PCM density compared with the cells expressing wild-type γ-tubulin-Flag at a similar level (Fig. 4*B* left), in which endogenous γ-tubulin expression was knocked down by RNAi. (Fig. 4*B*, right). Moreover, much more CDK5RAP2, an essential component of PCM, specifically its 51 to 100 aa sequence, was reported to bind γTuRC at MTOCs (25) and was observed to be associated with γTuRC in cells expressing Myc-c-Abl compared with cells expressing kinase-dead Myc-c-Abl(K290R) or cells treated with nilotinib (Fig. 4*C*). Accordingly, PCNT, an integral component of PCM, was diffusely distributed in a larger population of c-Abl/Arg-depleted cells and γ-tubulin(Y443F)-expressing cells than in wild-type cells (Fig. 4*D*). These results suggested that PCM proteins were recruited into the PCM at lower levels in the absence of c-Abl-mediated γ-tubulin phosphorylation. Further, the PCNT distribution and centriole structure were investigated by 3D-SIM super-resolution fluorescence microscopy. The proportion of cells containing 0-toroid PCNT increased from 10.7% in wild-type cells to 24.8% in c-Abl/Arg-depleted cells (Fig. 4*E*) and from 24.3% in cells ectopically expressing γ-tubulin-Flag to 47.1% in cells ectopically expressing γ-tubulin(Y443F)-Flag (Fig. 4*F*). These data suggested that c-Abl and Arg facilitate PCM formation through γ-tubulin phosphorylation and thus regulate centrosome assembly.

**Figure 5:**
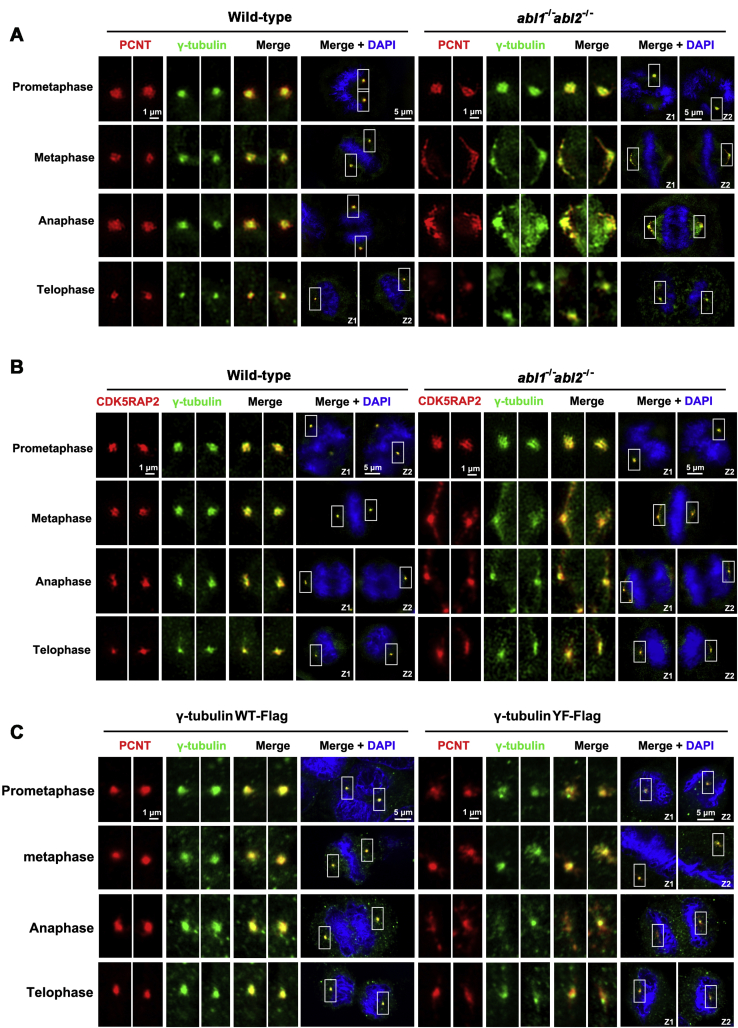
**c-Abl promotes mitotic centrosome assembly.***A* and *B*, immunofluorescence microscopy of wild-type or *abl1*^*−/−*^*abl2*^*−/−*^ U2OS cells stained with anti-γ-tubulin (*green*) and anti-PCNT (*red*) (*A*) or CDK5RAP2 (*red*) (*B*). *C*, immunofluorescence microscopy of γ-tubulin-Flag- or γ-tubulin(Y443F)-Flag-stably expressing MCF-7 cells stained with anti-γ-tubulin (*green*) and anti-PCNT (*red*). The nuclei were stained with DAPI (*blue*). Cells in different phases are shown. Centrosomes at different focal planes in each cell are shown in insets and are enlarged at left (Z1, Z2).

The centrosome increases in size to accommodate the recruitment of additional PCM proteins as cells transverse from interphase to mitosis (26). As expected, PCNT in mitotic cells was compact and globular but became expansive and irregular in c-Abl/Arg-depleted cells during mitosis (Fig. 5*A*). Similar findings were obtained with CDK5RAP2 (Fig. 5*B*) and in cells expressing γ-tubulin-Flag or γ-tubulin(Y443F)-Flag (Fig. 5*C*). These data suggested that the phosphorylation of γ-tubulin Y443 by c-Abl is essential for centrosome maturation during mitosis.

### c-Abl promotes MT assembly

**Figure 6:**
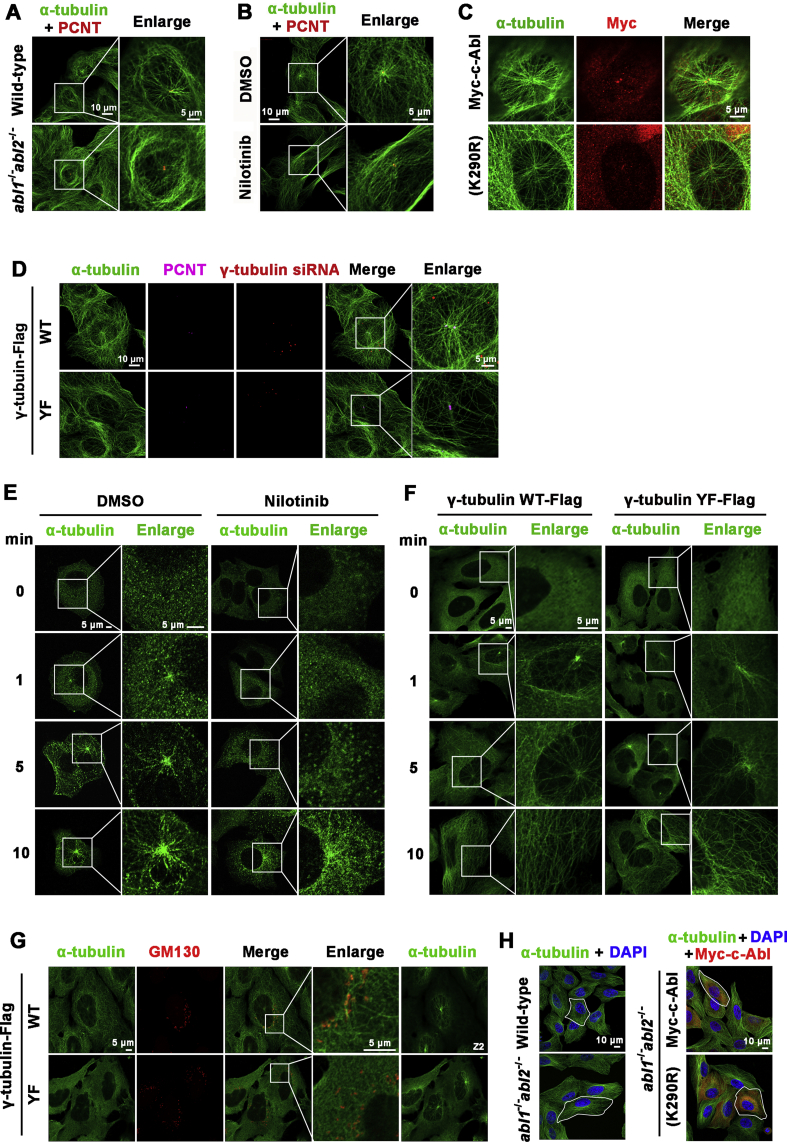
**c-Abl-mediated γ-tubulin phosphorylation regulates MT nucleation.***A*, immunofluorescence microscopy of wild-type or *abl1*^*−/−*^*abl2*^*−/−*^ U2OS cells stained with anti-α-tubulin (*green*) and anti-PCNT (*red*). *B*, U2OS cells treated with nilotinib (5 μM) or DMSO for 7 days were stained with anti-α-tubulin (*green*) and anti-PCNT (*red*). *C*, *abl1*^*−/−*^*abl2*^*−/−*^ U2OS cells were infected with adenovirus expressing Myc-c-Abl or Myc-c-Abl(K290R) and then stained with anti-α-tubulin (*green*) and anti-Myc (*red*). *D*, γ-tubulin-Flag or γ-tubulin(Y443F)-Flag U2OS cells in which endogenous expression was knocked down by RNAi (*red*) were stained with anti-α-tubulin (*green*) and anti-PCNT (*purple*). *E*, MCF-7 cells treated with nilotinib (5 μM) or DMSO for 7 h were incubated on ice for 2 h to depolymerize the MTs and then immersed in DMEM at 37 °C immediately. The cells were fixed at the indicated time points and stained with anti-α-tubulin. *F*, U2OS cells stably expressing γ-tubulin-Flag and γ-tubulin(Y443)-Flag were incubated on ice for 1 h and then analyzed as in (*E*). *G*, cells in (*F*) and treated as in (*F*), 5 min after 37 °C incubation, were fixed and stained with anti-α-tubulin (*green*) and anti-GM130 (*red*). The focal planes of centrosomal MTs are shown in the last panels marked with Z2. *H*, Wild-type or *abl1*^*−/−*^*abl2*^*−/−*^ U2OS cells were infected with Myc-c-Abl- or Myc-c-Abl(K290R)-expressing adenovirus and stained with anti-α-tubulin (*green*) and/or anti-Myc (*red*).

Next, we assessed the effect of Abl kinase-mediated γ-tubulin phosphorylation on MT organization. Centrosomal nucleated MTs were less dense in c-Abl- and Arg-depleted cells than in wild-type U2OS cells (Fig. 6*A*). Similar findings were obtained in cells treated with c-Abl and the Arg selective inhibitor nilotinib (Fig. 6*B*). Expression of c-Abl but not the dominant negative c-Abl(K290R) in c-Abl/Arg-null cells rescued the decreased density of MTs originating from the centrosome (Figs. 6*C* and S4*A*). Further, ectopic expression of RNAi-resistant γ-tubulin bearing the Y443F mutation in cells, in which endogenous expression of γ-tubulin was knocked down by siRNA, resulted in a significant decrease in centrosome nucleated MT formation compared with that in cells expressing a similar level of siRNA-resistant wild-type γ-tubulin (Figs. 6*D* and 2*B* right).

Further, the effect of c-Abl/Arg on MTs was analyzed by the MT regrowth assay. Consistent with the above findings, MT regrowth was significantly suppressed by nilotinib treatment (Fig. 6*E*) and by introducing γ-tubulin(Y443F) (Fig. 6*F*). Moreover, MT nucleation from the Golgi apparatus was also suppressed by the expression of γ-tubulin(Y443F) (Fig. 6*G*). Together, these results demonstrate that c-Abl and Arg play an important role in MT cytoskeleton formation. Consequently, compared with wild-type cells, c-Abl- and Arg-depleted cells were thinner and had a large surface contact area, which could be rescued by wild-type but not dominant negative c-Abl(K290R) (Figs. 6*H*, S4, *B* and *C*). These results further suggested that c-Abl/Arg played a role in the maintenance of cytoskeleton strength.

### c-Abl-mediated γ-tubulin phosphorylation regulates spindle formation

**Figure 7:**
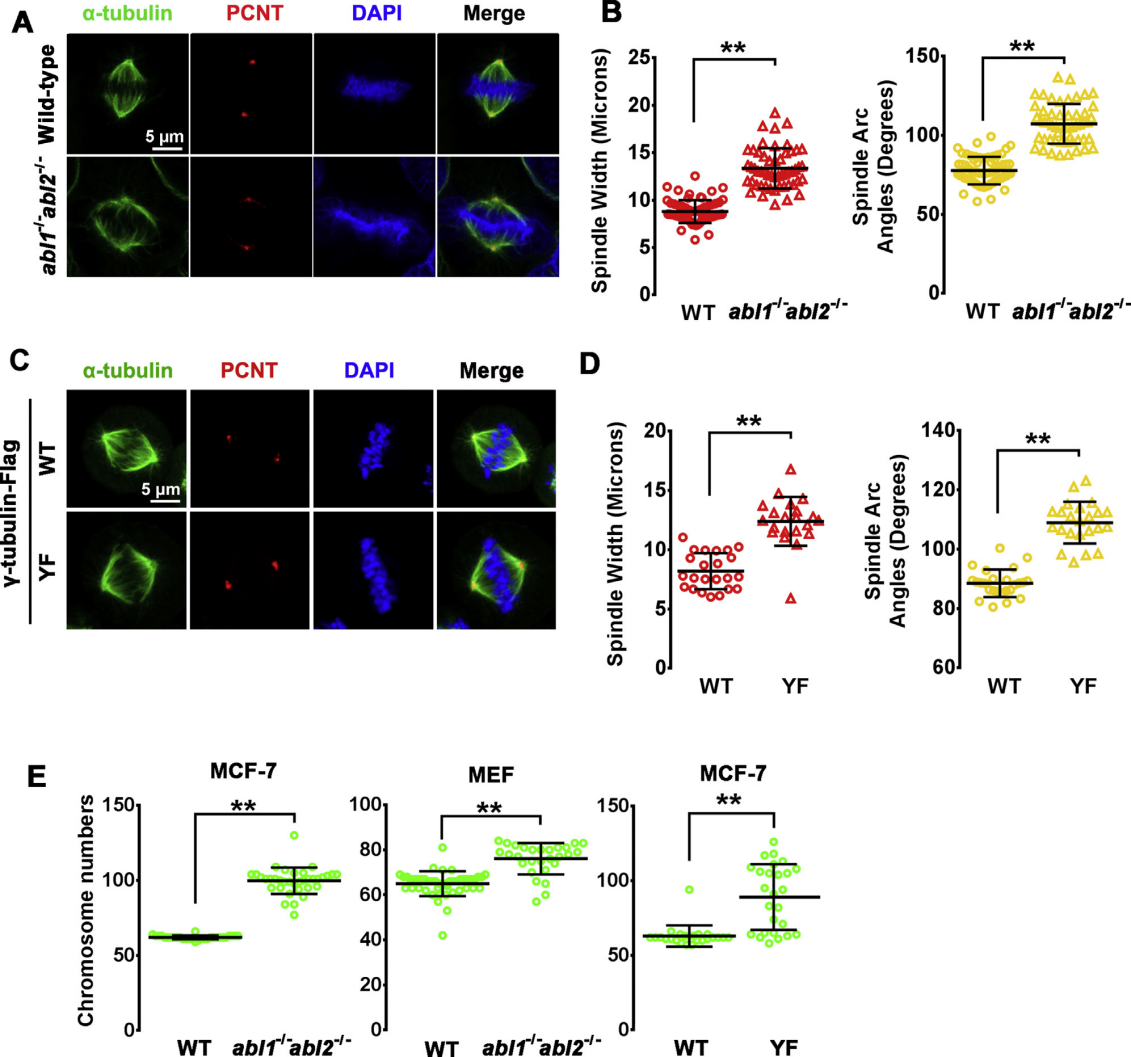
**c-Abl-mediated phosphorylation of γ-tubulin regulates spindle structure and mitosis.***A* and *B*, Wild-type and *abl1*^*−/−*^*abl2*^*−/−*^ MCF-7 cells were stained with anti-γ-tubulin (*green*) and anti-PCNT (*red*). Microscopy of metaphase cells (*A*), spindle width (*B*, *left*) and Arc angle (*B*, *right*) are presented. *C* and *D*, U2OS cells stably expressing γ-tubulin-Flag and γ-tubulin(Y443F)-Flag were stained with anti-γ-tubulin (*green*) and anti-PCNT (*red*). Microscopy of metaphase cells (*C*), spindle width (*D*, *left*) and Arc angle (*D*, *right*) are presented. *E*, chromosome numbers in wild-type and *abl1*^*−/−*^*abl2*^*−/−*^ MCF-7 cells (*left panel*), wild-type and *abl1*^*−/−*^*abl2*^*−/−*^ MEFs (*middle panel*), and γ-tubulin-Flag/γ-tubulin(Y443F)-Flag-expressing MCF-7 cells (*right*).

MTs play vital roles in the migration of chromosomes to opposite ends in mitotic cells *via* spindles during anaphase. As expected, spindles in metaphase were wider with a larger spindle angle in c-Abl/Arg-depleted cells and cells expressing γ-tubulin(Y443F) than in wild-type cells or cells ectopically expressing wild-type γ-tubulin (Figs. 7, *A*–*D* and S5*A* left). The chromosome congression index, based on the length to width ratio of the equatorial plate, was obviously decreased in c-Abl/Arg-depleted cells and γ-tubulin(Y443F)-expressing cells compared with wild-type cells and cells ectopically expressing wild-type γ-tubulin (Figs. 7, *A* and *C*, S5*A* right, S5, *B* and *C*). Consistent with these findings, significantly more chromosomes with a wider range were observed in c-Abl/Arg-depleted cells and γ-tubulin(Y443F)-expressing cells than in MCF-7 cells and wild-type MEFs (Figs. 7*E* and S5*D*). Collectively, these results suggested that c-Abl and Arg played important roles in proper chromosome segregation during mitosis.

## Discussion

Posttranslational modifications (PTMs) of tubulin, such as phosphorylation, acetylation, and polyglutamylation, play important roles in MT dynamics, while less is known about γ-tubulin modifications. Γ-tubulin of budding yeast is phosphorylated on Y445 in the DSYL motif at the C-terminus, and the replacement of this residue with aspartate (Y445D), which mimics phosphorylation, resulted in a temperature-sensitive mutant with increased numbers of MTs (27). However, this phenotype was not duplicated in *Tetrahymena thermophila* when the equivalent Y437 residue was mutated to D (28). In addition, S360 of yeast γ-tubulin was found to be phosphorylated by Cdk1 at spindle pole bodies, and the expression of γ-tubulin bearing the S360 to D mutation showed spindle defects involving changes in anaphase spindle MT dynamics (29). In mammals, γ-tubulin is reported to be phosphorylated on the S131 residue by the serine/threonine protein kinase SADB to control centrosome duplication (30). SADB also phosphorylates γ-tubulin on S385 to regulate the cellular localization of γ-tubulin and thereby control S phase progression (31). Several more serine or threonine phosphorylation sites (S32, S80, S129, S284, S364, T423, and S424) have been discovered by proteomic mass spectrometry in the PhosphoSitePlus database (www.phosphosite.org), but no functional evidence was reported. Γ-tubulin is also phosphorylated by protein tyrosine kinases, but to date, no tyrosine phosphorylation site has been identified. The Y92 to C mutation of γ-tubulin was observed in patients with malformations of cortical development (MCD) (32). Knocking in γ-tubulin Y92 C^/+^ in mice partially mimicked the human MCD phenotype, which might result from reduced MT dynamics (33). However, whether Y92 of γ-tubulin is phosphorylated is unclear. Several tyrosine kinases are reported to interact with γ-tubulin *in vitro*. In P19 embryonal carcinoma cells undergoing neuronal differentiation, Src and Fyn kinases bind γ-tubulin through their SH2 domains and phosphorylate it, regulating γ-tubulin interactions with tubulin dimers or other proteins during neuronal differentiation (34). In activated mast cells, Fyn-mediated phosphorylation of γ-tubulin complex proteins is involved in the regulation of MT formation in the initial stages (35).

In this study, for the first time, the nonreceptor tyrosine kinase c-Abl/Arg was demonstrated to bind γ-tubulin and to phosphorylate γ-tubulin on Y60, Y82, Y90, Y366, and notably, Y443 (the equivalent residue of Y445 in yeast γ-tubulin). Recombinant γ-tubulin expressed in *E. coli* was demonstrated to bind c-Abl directly, either by far-western blot (Fig. 1*F*) or SPR analysis (Fig. 1*G*). c-Abl inhibitor (AMN107) showed little if any effect on such direct interaction (Fig. 1*G*). These results suggested a moderate, kinase activity independent, direct interaction between c-Abl and γ-tubulin. Unlike other reported c-Abl substrates, γ-tubulin does not bind the c-Abl SH3 domain (Fig. 1, *I* and *J*), which may contribute to the fast-on/fast-off interaction profile (Fig. 1*G*). Since one of the disadvantages of the coimmunoprecipitation is the ability to detect weak interaction https://www.thermofisher.cn/cn/zh/home/life-science/protein-biology/protein-biology-learning-center/protein-biology-resource-library/pierce-protein-methods/co-immunoprecipitation-co-ip.html, no detectable c-Abl was observed in the anti-Flag immunoprecipitants in the presence of c-Abl inhibitors or kinase inactive c-Abl(K290R) (Fig. 2*E*). Like many other tyrosine kinase:substrate interactions, the association between c-Abl and γ-tubulin was significantly potentiated by tyrosine phosphorylation-mediated SH2 binding when active c-Abl was presented in the cells (Fig. 2*E*). Accordingly, γ-tubulin(Y443F) binds c-Abl less than wild-type γ-tubulin does, but the binding was significantly stronger than that of wild-type γ-tubulin in the presence of c-Abl inhibitors, since sites other than Y443 could also be phosphorylated by c-Abl (Fig. 2*E*). These results indicate that c-Abl associates with γ-tubulin and that this association is significantly potentiated by c-Abl-mediated phosphorylation of γ-tubulin. A detectable phosphorylation in *abl1/abl2* knockout cells suggested that γ-tubulin might also be phosphorylated by some other tyrosine kinases (Fig. 2*C*).

PCM protein recruitment to the daughter centriole occurs during mitosis through their transport along the centrosomal MTs on the parental centrosome by motor complexes (36). Therefore, new PCM formation relies on the successful MT assembly of the parental centrosome. The nucleation of MTs on the centrosome is dependent on the recruitment of γTuRC to the centrosome, and γTuRC itself is a PCM component that is tightly associated with scaffold proteins such as CDK5RAP2. In concert, c-Abl- and Arg-mediated phosphorylation of γ-tubulin promotes γTuRC assembly (Fig. 3) and facilitates PCM formation and centrosome maturation (Figs. 4 and 5), thus regulating MT organization in interphase cells (Fig. 6) and spindle formation in mitosis (Fig. 7). The PCM has an instructive function in centriole assembly and helps to stabilize the centriole and generate a functional centrosome (37). Accordingly, the triplet MT organization of the centriole seemed to be impaired in c-Abl/Arg-depleted cells and cells expressing γ-tubulin(Y443F) (Fig. 4, *A* and *B*), which suggested that c-Abl might influence the assembly of the centriole. Considering that nucleation of singlet MTs of the centriole requires the γTuRC complex localized in the PCM of the mother centrosome (38), we speculated that the PCM recruitment defect caused by c-Abl/Arg deletion caused centriole assembly impairment.

MTs play a central role in cellular architecture, cell cycle regulation, and intracellular transportation. As such, perturbations in MT assembly can lead to a range of defects in mitosis and other processes heavily dependent on the MT network. γ-Tubulin is highly expressed in the fetal brain and is important for neurodevelopment. The development of γ-tubulin knockout embryos stops at the morula/blastocyst stages in mice whose mitosis is defective due to disordered spindles (39). Errors in chromosome segregation during mitosis lead to chromosomal instability, which contributes to tumorigenesis and is thought to be a hallmark of human cancer (40). MTs have long been an important target for cancer treatment (41). In the central nervous system, MT dysfunction can lead to neurodevelopmental disorders and neurodegenerative disorders such as Alzheimer’s disease (AD) and Parkinson’s disease (PD) (42). In humans, γ-tubulin mutations may be related to MCD by destabilizing the α-helices or disrupting the GTP binding site of γ-tubulin and then impairing the assembly and/or functional integrity of the γTuRC (32). γ-Tubulin has also been found to aggregate in Lewy bodies in PD (43). Rotenone-treated neurons and astrocytes show aggregation of γ-tubulin and enlarged centrosomes. Disorganized centrosomes bring about cytoskeletal disturbances, disassembly of the Golgi apparatus and collapse of neuronal cells, which indicates that abnormal γ-tubulin underlies the etiopathogenesis of PD and related disorders (44).

c-Abl also participates in the regulation of the cell cycle, cancer, apoptosis, and the development and function of the central nervous system. Embryos in c-Abl- and Arg-depleted mice suffer from defects in neurulation and die before 11 days post coitum. The cytoskeleton in neuroepithelial cells shows gross alterations, but whether MT alterations occur is unclear (20). c-Abl overactivity is also observed in neurodegenerative pathologies, such as AD and PD (45, 46, 47, 48). The findings in this study that tubulin organization is regulated by Abl kinases provide a potential mechanism to understand these processes. c-Abl-regulated MT dynamics may also provide alternative approaches for the development of therapeutics, since c-Abl inhibitors such as STI571, nilotinib, and bafetinib have been successfully used in the treatment of blood and solid tumors and have shown efficacy in animal models of PD.

## Experimental procedures

### Cell culture and cell synchronization

Wild-type and *abl1*^*−/−*^*abl2*^*−/−*^ human breast cancer cells (MCF-7) and osteosarcoma cells (U2OS), human embryonic kidney cells (HEK293T), cervical cancer cells (HeLa), and wild-type and *abl1*^*−/−*^*abl2*^*−/−*^ mouse embryo fibroblast (MEF) cells were maintained in Dulbecco’s modified Eagle’s medium (DMEM, Gibco, Thermo Fisher) supplemented with 10% fetal bovine serum (FBS), penicillin (100 U/ml), and streptomycin (100 μg/ml). The cells were incubated at 37 °C with 5% CO_2_. In addition, the cells were treated with the indicated concentrations of STI571 or nilotinib.

### Plasmid construction, γ-tubulin silencing by siRNA, CRISPR/Cas9 mutagenesis of c-Abl and Arg, and transfection

Full-length γ-tubulin cDNA was amplified from an MCF-7 cDNA library by PCR. Flag-γ-tubulin or γ-tubulin-Flag plasmids were prepared by cloning the γ-tubulin gene (*tubg1*) with the sequence encoding the Flag tag into a pcDNA3.0-based vector (Clontech). The HA-GCP2 plasmid was purchased from Sino Biological Inc. The siRNA used to silence the γ-tubulin gene in this study was obtained from GenePharma, and the sequence was 5′-GCAAGGAGGACAUGUUCAAUU-3’. An siRNA-resistant γ-tubulin-expressing plasmid was generated by introducing two silent synonymous mutation mutations in the siRNA-targeted sequence (5′-GCAAGGAGGACATGTTCA-3′ mutated to 5′-GCAAGGAAGATATGTTCA-3′) using the Muta-Direct Site-Directed Mutagenesis Kit (SBS Genetech). Flag-γ-tubulin and γ-tubulin-Flag with Y→F mutations were also prepared using a direct mutagenesis strategy.

Protein-expressing plasmids were transfected into HEK293T cells using Lipofectamine 2000 according to the manufacturer’s protocol (Life Technologies, Inc). U2OS and MCF-7 cells stably expressing wild-type γ-tubulin-Flag or γ-tubulin(Y443F)-Flag were generated by transfection of the respective plasmids and puromycin screening. The siRNAs were transfected with TransIT-X2 (Mirus Bio) according to the manufacturer’s instructions.

*abl1* (encoding c-Abl) and *abl2* (encoding Arg)-knockout cells were generated by the CRISPR/Cas9 system. sgRNAs (sgRNA-*abl1:* 5ˊ-TGTGATTATAGCCTAAGACC-3ˊ and sgRNA-*abl2:* 5ˊ-AGTTCGCTCTAAGAATGGGC-3ˊ) were cloned into the pSpCas9(BB)-2A-Puro vector (Addgene plasmid ID: 48,139), after which the resulting plasmids were cotransfected into U2OS cells using Lipofectamine 3000, and the cells were screened with puromycin. Stable clones selected 48 h after transfection were identified by genomic sequencing and immunoblotting with specific antibodies.

### Immunoprecipitation and immunoblotting

Cells were lysed using cell extraction buffer (50 mM Tris-HCl [pH 7.5], 150 mM NaCl, 1 mM EDTA, 1% Nonidet P-40, and protease inhibitor cocktail from Roche). Soluble proteins were subjected to immunoprecipitation with anti-γ-tubulin (Santa Cruz), anti-Flag (Sigma-Aldrich), anti-p-Tyr (Merck), anti-GCP5 (Santa Cruz) antibodies, or anti-mouse IgG (Sigma-Aldrich). An aliquot of the total lysate (5%, v/v) was included as the input. Protein samples were separated by SDS-PAGE and transferred onto PVDF membranes. Anti-γ-tubulin (Sigma-Aldrich), anti-c-Abl (Santa Cruz), anti-Arg (Santa Cruz), anti-GCP5 (Santa Cruz), anti-GCP2 (Novus), and anti-GCP3 (Proteintech) antibodies were used as primary antibodies to detect the corresponding proteins. Horseradish peroxidase (HRP)-conjugated anti-mouse IgG (Sigma-Aldrich) or anti-rabbit IgG (Sigma-Aldrich) was used as the secondary antibody. HRP-conjugated anti-p-Tyr (Merck), anti-Flag (Sigma-Aldrich), anti-Myc (Sigma-Aldrich), anti-GST (Santa Cruz), and anti-β-actin (Sigma-Aldrich) antibodies were used directly to detect the corresponding proteins. The protein bands were visualized using an ECL detection system (Millipore) at the final step with a chemical imaging analyzer (GE Healthcare).

### Confocal immunofluorescence microscopy and 3D structured illumination microscopy (SIM)

The adherent cells cultured on coverslips were fixed in 4% paraformaldehyde for 20 min at room temperature, followed by membrane permeabilization using 0.2% Triton X-100 in PBS for 10 min. For MT staining, prefixation was performed before the fixation step by adding a half volume of preheated 4% paraformaldehyde and incubating for 10 min at 37 °C. Then, the samples were blocked with 1% FBS for 1 h and incubated with anti-α-tubulin (Sigma-Aldrich), anti-PCNT (Abcam), anti-c-Abl (Sigma-Aldrich), anti-MG130 (Proteintech), anti-γ-tubulin (Sigma-Aldrich), anti-CDK5RAP2 (Thermo Fisher), or anti-Myc (Proteintech) antibody for 1 h at room temperature, followed by incubation with FITC-, TRITC-, or Alex647-conjugated anti-mouse IgG or rabbit IgG secondary antibodies for another 1 h at room temperature. The nuclei were stained with DAPI at the final step. For confocal image acquisition, the slides were viewed by a Zeiss (LSM 800) confocal microscope using a 63 × oil objective and laser lines at 488 nm, 561 nm, and 647 nm wavelength for excitation.

For SIM image acquisition, the slides were viewed on an N-SIM System (Nikon) equipped with a 100 × 1.49 NA Apo oil objective lens (Nikon). Z-sections with a Z-distance spanning the entire volume of centrosomes were acquired in 3D SIM mode, generating 15 images per plane as a raw image, which was further computationally reconstructed to generate a super-resolution image using NIS-Elements software (Nikon). Centrosomes viewed as cross sections of the PCM were selected for analysis.

### MT regrowth assay

U2OS or MCF-7 cells cultured on coverslips were placed on ice for 1 h or 2 h, respectively, to depolymerize MTs. MT regrowth was allowed by incubating the cells in prewarmed medium at 37 °C for the indicated times. Then, the cells were immediately immersed in methanol for 10 min at room temperature for fixation and subjected to immunostaining with an anti-α-tubulin antibody.

### Transmission electron microscopy (TEM)

Cells were washed with PBS, fixed with 2.5% glutaraldehyde, and then prestained with osmium tetroxide. Eighty-nanometer-thick serial sections were then cut and stained with uranyl acetate and lead citrate. Images were acquired with a transmission electron microscope (Hitachi, H-7650) operating at 80 kV.

### Gel filtration chromatography and sucrose gradient centrifugation of cytosolic γTuRC

Cells were washed with HBS (50 mM HEPES, pH 7.4, and 150 mM NaCl) and then lysed at 4 °C in HBS supplemented with 1 mM DTT, 1 mM EGTA, 1 mM MgCl_2_, 0.25 mM GTP, 0.5% Triton X-100, and protease inhibitor cocktail. The lysates were precleared by spinning in a microcentrifuge at 12,000*g* for 10 min and were further clarified by centrifugation at the same speed for 20 min. Then, 0.5 ml of the lysates was layered in a Superose-6 column (GE Healthcare) in HB buffer, and 0.5-ml or 1-ml fractions were collected and subjected to SDS-PAGE and immunoblotting.

CDK5RAP2 in complex with γTuRC was isolated by immunoprecipitation from Flag-GFP-CDK5RAP2(51-100)-transfected HEK293T cells. Then, the Flag-bound proteins were eluted by incubating the beads for 30 min with 0.2 mg/ml Flag peptide in HBS buffer. The eluate was subjected to sucrose gradient (5–50%) centrifugation. After centrifugation, each fraction was resolved by SDS-PAGE and subjected to immunoblotting.

### Protein-binding assays

In GST pull-down experiments, cell lysates were incubated for 2 h at 4 °C with approximately 5 μg of purified GST or GST fusion protein conjugated with glutathione beads. The absorbates were then washed with lysis buffer and subjected to SDS-PAGE followed by immunoblotting analysis. An aliquot of the total lysate (5%, v/v) was included as a loading control in SDS-PAGE.

In far-western (direct) binding assays, immunoprecipitates were separated *via* SDS-PAGE and then blotted onto PVDF membranes. The membranes were subsequently incubated with purified GST or GST fusion proteins for 2 h at room temperature. The binding of the GST fusion proteins to PVDF membranes was probed using an anti-GST antibody.

### *In situ* proximity ligation assay (PLA)

The Duolink *in situ* PLA (Sigma-Aldrich, MO) was applied to detect the interactions between γ-tubulin and c-Abl in HeLa cells stably expressing GFP-H2B. Briefly, cells on glass coverslips were fixed with 4% paraformaldehyde and then permeabilized with 0.2% Triton X-100 in PBS. After blocking, the cells were incubated with antibodies against γ-tubulin (mouse monoclonal antibody, Sigma-Aldrich) and c-Abl (from rabbit, Sigma-Aldrich) and then incubated with oligonucleotide-labeled anti-mouse IgG and anti-rabbit IgG according to the manufacturer's instructions for PLA. The red fluorescent spots generated by the DNA amplification-based reporter system were detected with a Zeiss (LSM 800) confocal microscope.

### SPR assay

Abl1 (ab136358, abcam) was immobilized on CM5 sensor chips (Biacore, GE) at a level of ∼800 response units (RUs) using a Biacore T200 (Biacore, GE) and running buffer composed of 0.01 M HEPES (pH 7.4), 0.15 M NaCl, 3 mM EDTA, and 0.05% Tween-20. Serial dilutions of γ-Tubulin (Ag23913, Proteintech) were flown through at concentrations ranging from 25 to 800 nM. Steady-state affinity was fitted using Biacore Evaluation Software (Biacore, GE).

### LC-MS/MS analysis

Anti-Flag immunoprecipitates prepared from lysates of HEK293T cells transfected with Flag-γ-tubulin and Myc-c-Abl were resolved by SDS-PAGE, after which the protein bands were excised and subjected to trypsin digestion. LC-electrospray ionization-MS/MS-resolved peptides were analyzed using a Q-TOF2 system (Micromass), and the data were compared against the SwissProt database using the Mascot search engine (http://www.matrixscience.com) for phosphorylation modification.

### Calculation of changes in binding free energy

The binding free energy changes in γ-tubulin with GCPs were estimated by the FoldX program using a full atomic description of the structure of the proteins. The different energy terms included in FoldX have been weighted using empirical data obtained from protein engineering experiments. Molecular structure illustrations were generated with PyMOL Molecular Graphics Software (www.pymol.org). The atomic coordinates (PDB accession code: 6v6s) for the cryo-EM structure of native human γTuRC were obtained from the Protein Data Bank. The error margin of FoldX is approximately 0.5 kcal/mol, so changes in that range are considered as insignificant.

### Measurements and statistical analysis

The intensities of immunoblotting bands were measured using ImageJ software (NIH). Statistical analyses were carried out using GraphPad Prism (GraphPad Software). At least three independent experiments were carried out to generate each dataset, and statistical significance in each case was calculated using Student’s *t* test as indicated in the figure legends. *p* < 0.05 and *p* < 0.01 were considered statistically significant and are indicated by ∗ and ∗∗, respectively.

## Data availability

This study did not generate/analyze [datasets/code].

## Supporting information

This article contains supporting information.

## Conflict of interest

The authors declare that there is no conflict of interest with the contents of this article.
