# Supplementary material for: c-Abl kinase-mediated phosphorylation of γ-tubulin promotes γ-tubulin ring complexes assembly and microtubule nucleation
Source: J Biol Chem. 2022 Feb 26;298(4):101778. doi: 10.1016/j.jbc.2022.101778 (PMC8980629; doi:10.1016/j.jbc.2022.101778)
Supplement: Supplemental Figures S1–S5 [file mmc1.pdf]

Supplemental Figures

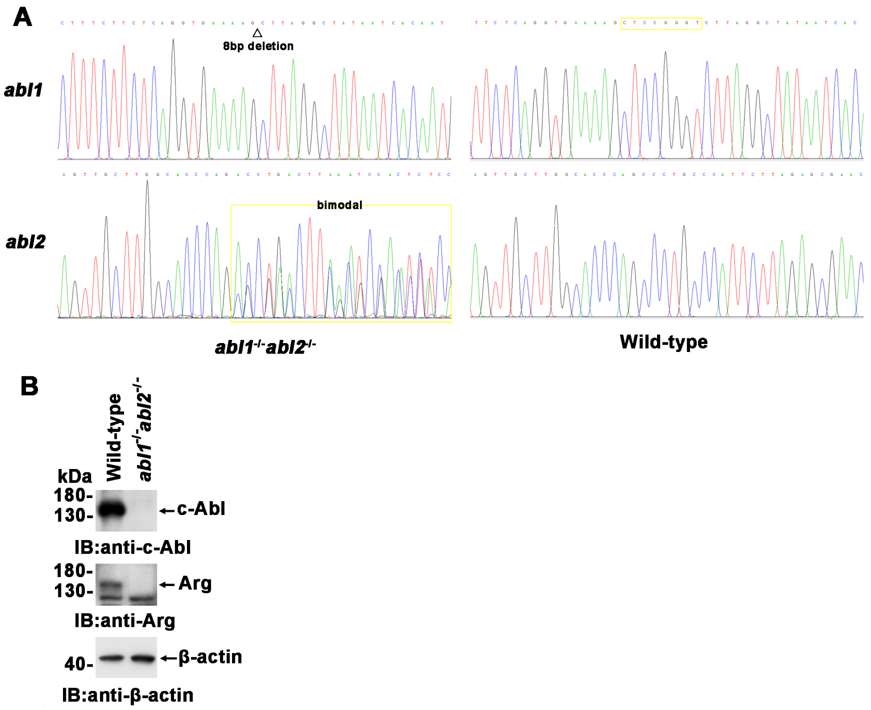

Figure S1. Generation of c-Abl and Arg double-knockout cell lines. (A). c-Abl and Arg double-knockout (DKO) MCF-7 cell lines were generated by the CRISPR/Cas9 strategy. The sequences of the DNA editing regions were shown. B. c-Abl and Arg DKO cell clones were confirmed by immunoblotting using anti-c-Abl and Arg antibodies.

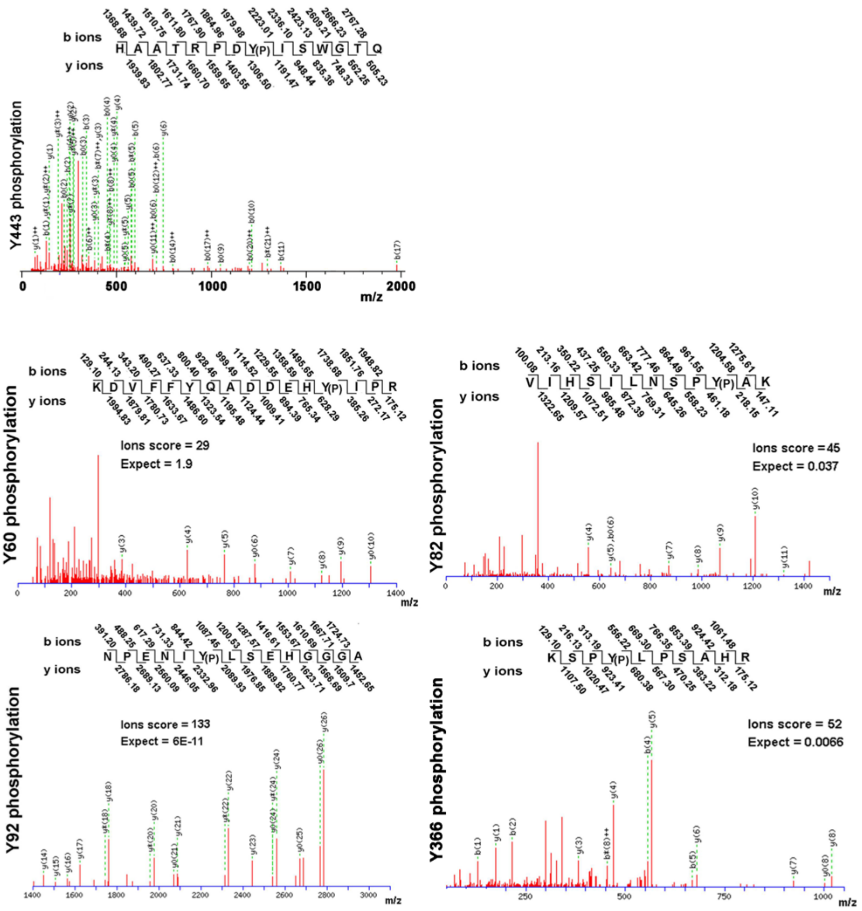

Figure S2. Identification of phosphorylation sites on γ-tubulin mediated by c-Abl. Anti-Flag immunoprecipitates from HEK293T cells cotransfected with Myc-c-Abl and Flag-γ-tubulin were resolved by SDS-PAGE, and the Flag-γ-tubulin band was excised and subjected to LC-MS/MS analysis. Monophosphorylated peptide containing PO_3-modified tyrosines are shown, and the

peptide sequences are shown at the top of the panels.

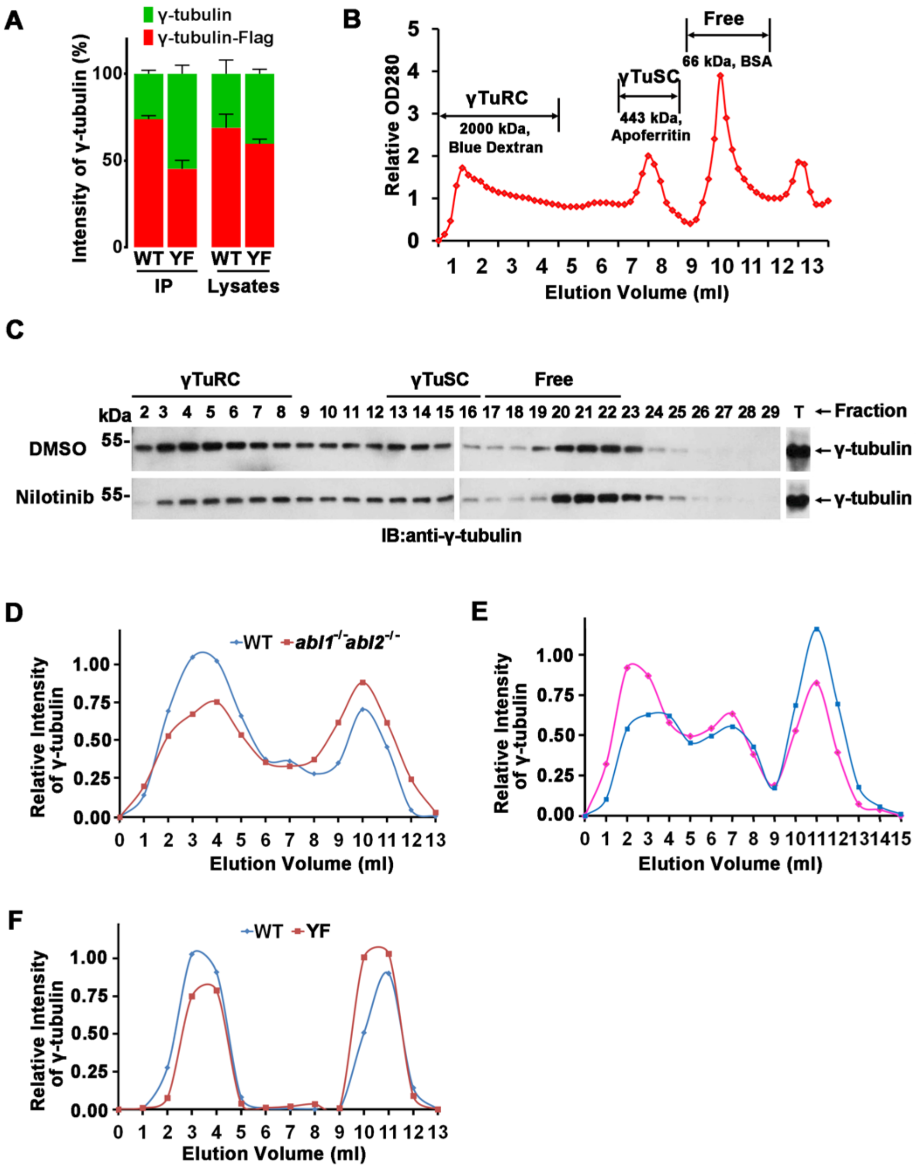

Figure S3. c-Abl promotes γTuRC assembly by phosphorylating γ-tubulin. (A). The ratios of ectopically expressed γ-tubulin and endogenous γ-tubulin calculated by the intensity of bands in Figure 3D. (B). A mixture containing 1 mg blue dextran, 1 mg apoferritin and 1 mg BSA was fractionated by gel filtration chromatography. (C) Lysates of MCF-7 cells treated with or without nilotinib were fractioned by gel filtration, and a 0.5-ml fraction was collected and analyzed by immunoblotting with anti-γ-tubulin. (D-F) Relative densities of the bands from Figure 3E(D), Figure S3C(E) and Figure 3F(F), which was normalized with total lysates, were quantified with ImageJ.

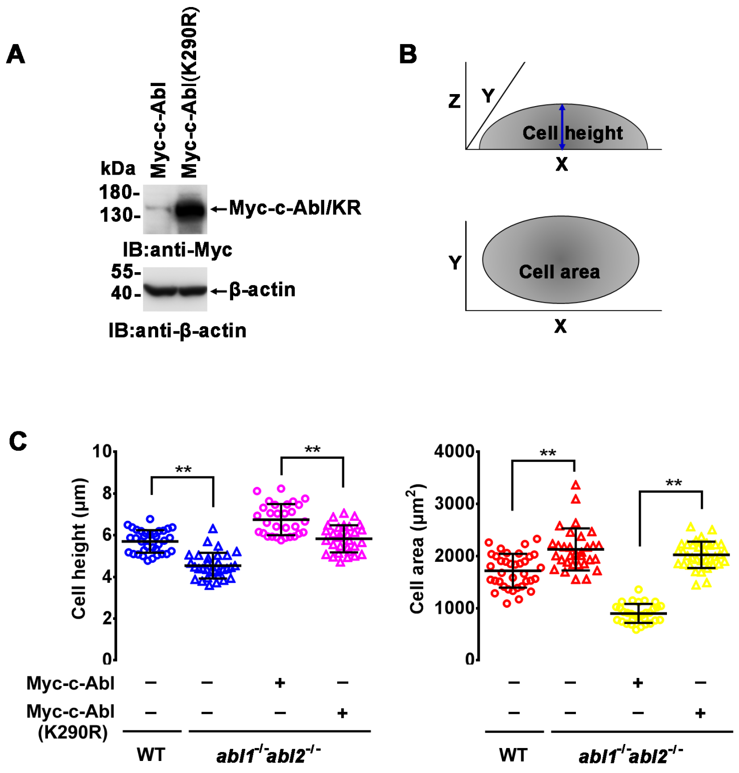

Figure S4. c-Abl-mediated γ-tubulin phosphorylation promotes MT nucleation. (A). U2OS cells were infected with Myc-c-Abl and Myc-c-Abl(K290R)-expressing adenovirus for 2 days, as shown in Figure 6C, and their expression was determined by immunoblotting. (B) Cartoon illustrations of the cell height and area shown in Figure 6I. (C) The distributions of the cell height (left) and cell area (right) of the indicated cell lines. Data from individual cells and the mean±SD are presented.

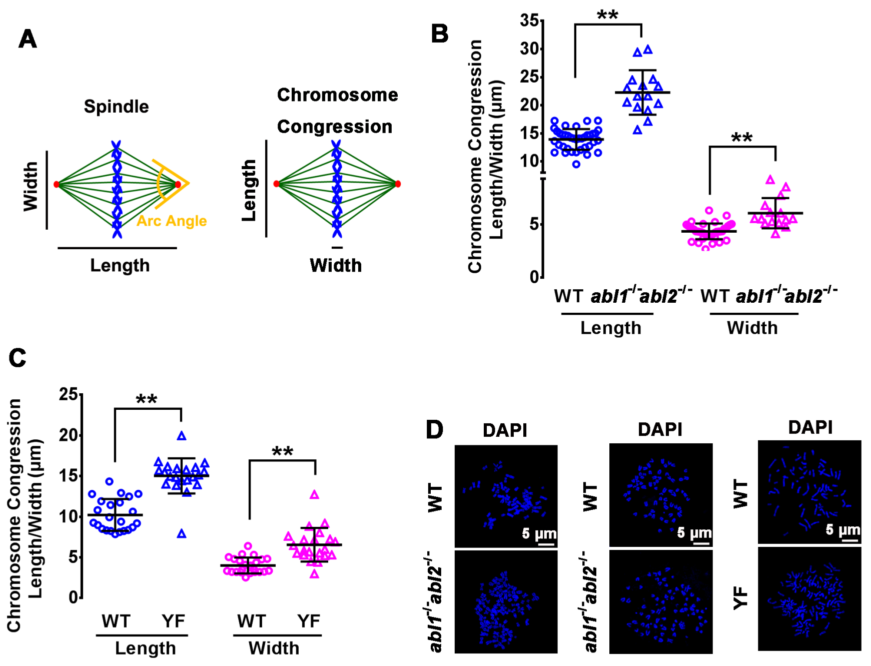

Figure S5. c-Abl-mediated γ-tubulin phosphorylation regulates spindle structure and mitosis. (A) Diagrams of spindle length, width and Arc angle. (B) Chromosome congression length and width in mitotic wild-type or *abl1*$^{-/-}$*abl2*$^{-/-}$ MCF-7 cells. (C) Chromosome congression length and width in mitotic γ-tubulin-Flag- or γ-tubulin(Y443F)-Flag-expressing U2OS cells. (D) Karyotype of wild-type and *abl1*$^{-/-}$*abl2*$^{-/-}$ MCF-7 cells (left), wild-type and *abl1*$^{-/-}$*abl2*$^{-/-}$ MEFs (middle panel), and γ-tubulin-Flag and γ-tubulin(Y443F)-expressing MCF-7 cells (right panel).
